# Supplementary material for: The expanding movement of primary care physicians operating at the first line of healthcare delivery systems in sub-Saharan Africa: A scoping review
Source: PLoS One. 2021 Oct 22;16(10):e0258955. doi: 10.1371/journal.pone.0258955 (PMC8535187; doi:10.1371/journal.pone.0258955)
Supplement: S1 Text — (PDF) [file pone.0258955.s001.pdf]

A new bird in the landscape of Primary Health Care: a scoping review on First Line Medical Doctors in Sub-Saharan Africa

Version of April 2019

Background

The celebration of the 40th years of the Alma Ata declaration has been the occasion to recognize that Primary Health Care (PHC) and its values are more than relevant is our current world(1,2). An important dimension of Primary Health Care is the first contact care referred to as "primary care(3)". Barbara Starfield defined primary care in 1994 as "the first level of a health care system where people present their health problems and where the majority of the population's curative health needs, health promotion and preventive health needs are satisfied (4)". Achieving a good primary care requires a workforce that is adequately trained, motivated and distributed, with appropriate allocations of tasks and responsibilities(2,5).

In Sub-Saharan Africa, the provision of primary care relies mainly on non-physician health professionals (3). Indeed, in many African health policies, the task-shifting from medical doctors to nurses is a widely adopted approach. This is due to doctors' shortage and evidence that task-shifting can be effective for the management of some diseases and for improving access to health care (6,7). However, a growing number of doctors is graduating from the medical schools (8). This situation, combined to the low capacity of the State to enroll them and the development of the private sector led to a growing settlement of medical doctors for the provision of primary care (8–10). Little is known about the current functioning and roles of these "first line medical doctors" in Sub-Saharan Africa, on the tensions and even disruptions their functioning may create in existing health care delivery systems, on their current performance in terms of providing quality and equitable primary health care, and on the implications in terms of systems design and management for that potential to effectively realize.

This scoping review aims at contributing to gaining this knowledge by providing an overview of the current literature on First Line Medical Doctors (FLMD) in Sub-Saharan Africa and identifying the knowledge gaps.

In 2003, Van Dormael and Dugas studied the experience of Mali and South Africa in constructing family medicine(10). But, to the best of our knowledge, this review will be the first to pull together the existing knowledge on First Line Medical Doctors in Sub-Saharan Africa.

This review is the first phase of a PhD research project aiming at studying in detail the existing experiences in Benin with FLMDs' practices, and eventually propose policies with regards to their role(s) in achieving more performant PHC systems.

Objectives of the literature review

General objective

The general objective is to map the existing knowledge on First Line Medical Doctors (FLMD) in Sub-Saharan Africa and to identify the key issues in this area.

Specific objectives and research questions

a. Determine the various types of medical Doctors operating at the level of first-line in SSA
b. Map the key dimensions that have been studied on FLMD in Sub-Saharan Africa (training, resources, services delivery, leadership and government arrangements ,etc.)
c. Determine the key issues related to FLMDs' practice in Sub-Saharan Africa

Methodology

Type of literature review

We will perform a scoping review. According to Mays and al (11)a scoping review "aims to map rapidly the key concepts underpinning a research area and the main sources and types of evidence available". Therefore, this methodology suits well with the objective of this review: to map the key dimensions that have been studied on FLMD in Sub-Saharan Africa and determine the key issues related to their practice. In this work, we will follow the six steps proposed by Arksey and O'Malley in 2005(12) for conducting a scoping review:

- identification of the review question,
- identification of relevant studies,
- study selection,
- charting the data
- collating, summarising and reporting the results,
- consultation of stakeholders in order to inform and validate the findings.

We will also be guided by previous scoping reviews protocols ((13,14) and various methodology guides on literature reviews, especially approach proposed by the Johanna Briggs institute (15,16).

A scoping review protocol has been developed and discussed among all the authors.

Identification (and framing) of the review question:

The review question is the following:

"What are the main characteristics and key issues of First-Line Medical Doctors' practice in SSA?"

We chose to keep this question quite broad at this stage as this is a scoping review. Arksey and O'Malley (2005) recommended indeed to "maintaining (at the outset of a scoping review) a wide approach in order to generate breadth of coverage". Nonetheless, it is important to clearly define the key terms in the research question(12).

In our research question we need to clearly define the expression "First-Line Medical Doctor":

We define “First Line Medical Doctor” (FLMD) as a medical doctor working (permanently or in an intermittent way) at the first-line of healthcare delivery. So, a FLMD can work or intervene at first-line health facilities. But a FLMD can also work directly in the community.

To better fame this research question, we will used the “PCC” approach proposed by the Joanna Briggs Institute to structure scoping reviews questions(15).

Population: Medical Doctors

Concept: First-Line practice OR Primary Care

Context: Sub-Saharan Africa

For the “concept”, we also used Primary Care because the core function of the first-line of healthcare delivery is to provide primary care. So, in the search strategy we should also use the expression “primary care”

The key words in this table will be used for the search in PubMed. They will be used (with relevant adaptations) for the search in other databases

Table 1: Framing of the literature review research question using a PCC approach and the key words

PCC	“Common” term	Key words to be used in the search strategy
Population	Medical Doctors	"Physicians"[Mesh] OR "medical doctor" OR doctor OR “family physician*” OR “family doctor*”OR “primary care physician*” OR “primary care doctor*” OR “general doctor*” OR “general practitioner*” OR GP OR “medical officer”
Concept	First line practice	"Primary Health Care"[Mesh] OR “primary health care” OR "Community Health Services"[Mesh] OR "Community Health Centers"[Mesh] OR "primary care" OR "first line" OR "first level" OR "health center*" OR “general practice” OR “family medicine” OR “family practice” OR “private” OR "Private Practice"[Mesh] OR "Private Sector"[Mesh] OR "Private Facilities"[Mesh] OR “front line” OR “front-line”
Context	Sub-Saharan	"Sub Saharan Africa" OR "Africa South of the Sahara"[Mesh] OR Angol* OR Benin* OR Botswana OR “Burkina Faso” OR Burkinabe* OR Burundi OR Cameroon* OR Cameroun* OR “Cape Verde” OR “Cape Verdean” OR “Cabo Verde” OR “Central African Republic” OR Chad* OR Tchad* OR Comoros OR Comorian OR “Democratic Republic of Congo” OR “Republic of Congo” OR Congo OR Congolese OR RDC OR DRC OR Zaire OR “Republique Democratique Congo” OR “Côte d’Ivoire” OR “Republic of Côte d’Ivoire” OR “Ivory Coast” OR Ivorian OR Djibouti* OR “Equatorial Guinea” OR Guinea* OR Eritrea* OR Ethiopia* OR Gabon* OR Gambia* OR Ghana* OR Guinea OR “Guinea-Bissau” OR Kenya* OR Lesotho OR Liberia* OR Libya* OR Madagascar OR Malawi* OR Mali OR Malian OR Malien OR Mauritania* OR Mauritius OR Mauritian OR Mozambique OR Mozambican OR Namibia* OR Niger* OR Nigeria* OR Rwanda* OR “Sao Tome Principe” OR “Sao Tomean” OR Senegal* OR Seychell*

		OR “Sierra Leon*” OR Somali* OR “South Africa” OR “South African” OR Sudan* OR Swaziland Or Swazi OR Eswatini OR Tanzania* OR Togo* OR Uganda* OR Zambia* OR Zimbabwe*

Identification of relevant studies

Sources

The following sources will be used.

- **Electronic Data bases mainly for peer-reviewed publications***:* MEDLINE (through PubMed), COCHRANE Library, “Banque de Donnees en Sante Publique” , Web of Science, Health system evidence.
- **Experts and websites mainly for grey literature**: World Organization of Family Doctors (WONCA), “Santé Sud”, Primary Care & Family Medicine Education Network (PRIMAFAMED), Belgian Development Agency, (ENABEL) World Health Organization (WHO), Primary Health Care Performance Initiative (PHCPI), European Forum of Primary Care, Institute of Tropical Medicine (ITM), etc.
- **Reference lists (snowballing):** Other literature sources from the bibliographies of relevant papers

Search strategy

Our search strategy will use the key words identified in table 1. We will first search on MEDLINE using both Medical Subject Headings and free text terms. The search strategy used on MEDLINE is:

This strategy will be adapted and used for the other databases using thesaurus words as well as free text terms.

The search will not be limited by year of publication nor by study design. However, it will be limited to English and French languages.

At this step, we will ask for the support of an experienced researcher in information science which is one of the co-authors of this article (ZB).

Study selection

All the search results will be compiled in a database using the reference manager software Mendeley. Duplicates will be removed. Two reviewers will then assess independently the titles and the abstracts by using the inclusion and exclusion criteria. Full texts will be read and assessed for the citations that meet the criteria and those for which there is a doubt. The full texts that are relevant to answer the research question will be included in the review. At each step, the two reviewers will compare their selections in order to check for discrepancies. In that case, agreement will be met through discussion between the two reviewers. In case of persistent disagreement, the opinion of the senior supervisors of the review will be asked.

Based on the review question and preliminary search on PubMed with a quick screening of the results yield, the following inclusion and exclusion criteria were defined:

<u>Inclusion criteria</u>:

Type of documents

- Research papers (peer-reviewed articles, reports, etc.)
- Case studies /reports of interventions

Content

- Articles talking of medical doctors at first line or primary care setting or in the community (in the public or private sector)
- Articles about task-shifting in primary care and comparing the results achieved by nurses with the one achieved by doctors

Exclusion criteria:

Type of documents

- Opinion papers
- Conferences/workshop reports

Content

- Articles only relating the practice of medical doctors (including General Practitioners) in hospital care settings
- Articles only relating the practice of medical doctors (including General Practitioners) only taking care of specific populations (HIV patients for instance)
- Articles only relating the practice of non-physicians (nurses for instance), without comparing it to medical doctors.
- Guidelines are excluded. Even in the case that they are addressed to FLMD, they don't give any information on their practice
- Study related to PHC workers or PHC facilities in general without precising whether the study population includes doctors or not
- Studies that does not include doctors working in Sub-Saharan Africa

These criteria may be refined after as we get more familiar with the existing literature on FLMD.

Indeed, in April 2019 we added a few more exclusion criteria, in order to focus on the actual practice of FLMDs. We further excluded:

- Studies only assessing the knowledge of the FLMDs without assessing their actual practice
- Studies reporting a specific experience in an controlled environment . For instance, we excluded a study reporting the results a training programme among primary care providers to offer brief behaviour change counselling on risk factors for non-communicable diseases in South Africa, because this programme happened in a controlled environment and was not really representative of FLMD's usual practice.

Data charting

FLMD operate into a (local) health system and the different elements of this health system as well as their interrelations will influence the way FLMDs work and the results achieved. Therefore, we think

that a health system framework will help us to organize the existing knowledge on FLMD. In this review, we will use the Health Systems Dynamics (HSD) framework (figure 1). This framework will guide the data extraction from the study.

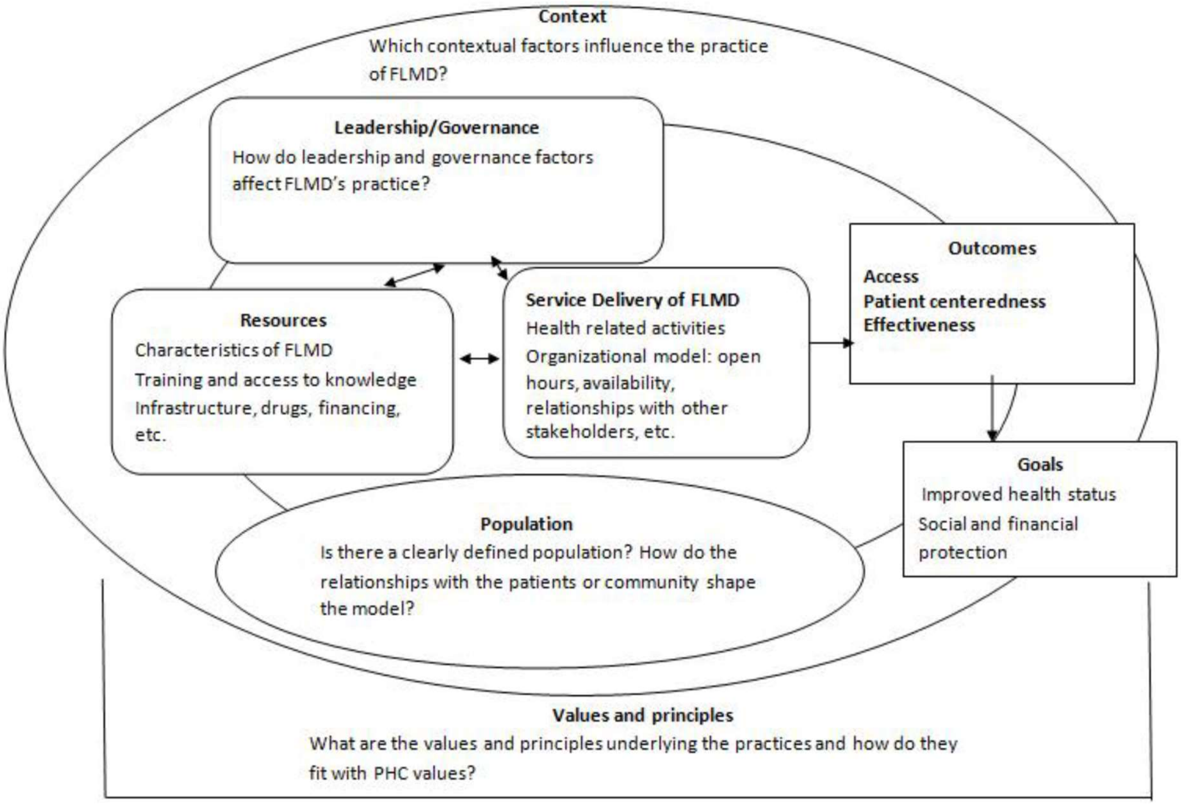

Figure 1: FLMD and the health systems dynamics framework (adapted from Van Olmen and al)

For the selected paper, each reviewer will independently extract relevant information using an excel sheet (the data charting form). This excel sheet is based on the research question, health systems dynamics framework. The data to be extracted (see annex 1)relate to :

- Title of the study
- First author
- Year of Publication
- Country
- Language
- Names used for the FLMDs in the paper
- Study design
- Objectives of the paper
- Governance
    - Workplace ownership (public, private)
    - Policy guidance and regulation
    - Other governance aspects
- Resources
    - Pre-graduate training
    - Post-graduate training

  - Access to knowledge and information
  - Support received
  - Funding, equipment, infrastructures and supply
- Service delivery
  - Role assigned to FLMDs
  - Activities performed
  - Relationships of FLMDs with other workforce
- Relationship with the community (including presence of any assigned population)
- Context
- Values /principles, organizational culture
- Outputs/outcomes
  - Access to care (Financial access, Geographic access, other aspects of access)
  - Patient centeredness
  - Comprehensiveness
  - Coordination
  - Technical Quality of care
  - Continuity
  - Health outcomes
- Additional findings
- Comment on the study

Differences in data extraction between the two reviewers will be discussed and submitted to the supervisors in case of disagreement. The data extracted will then be merged

Collating, summarizing and reporting the results

Using the approach proposed by Arksey and O'Malley, we will provide first a numerical summary of the number of studies and the study settings.

We will then use qualitative content analysis technics to summarize the data extracted in the data charting form, as recommended by Levac and al (17) . Each reviewer will then independently code the data extracted. The two reviewers will then discuss and refine the data analysis together. The main themes will be identify and the findings will be organized according to these themes. The resulting summary will be presented to the rest of the review team for refining the analysis.

Consultation of stakeholders in order to inform and validate the findings

The preliminary findings will be discussed with experts working in the field of Primary Health Care, Family medicine and other related domains. This consultation will help not only to validate our findings but also to have additional information and analysis from the experts experience. This consultation will actually start from the beginning in order to have access to the grey literature.

References

1. Hone T, Macinko J, Millett C. Revisiting Alma-Ata: what is the role of primary health care in achieving the Sustainable Development Goals? Lancet. 2018 Oct;392(10156):1461–72.

2. Global Conference on Primary Health Care. Declaration of Astana. 2018 p. 1–12.

3. World Health Organization (WHO). Primary Health Care: Now More Than Ever. 2008.

4. Expert Panel on Effective Ways of Investing in Health. Definition of a frame of reference in relation to primary care with a special emphasis on financing systems and referral systems. 2014;(February):1–66.

5. Pettigrew LM, De Maeseneer J, Padula Anderson M-I, Essuman A, Kidd MR, Haines A. Primary health care and the Sustainable Development Goals. Lancet. 2015;386:2119–21.

6. Eyal N, Cancedda C, Kyamanywa P, Hurst SA. Non-physician Clinicians in Sub-Saharan Africa and the Evolving Role of Physicians. Int J Heal Policy Manag. 2015;5(3):149–53.

7. Primary Health Care Performance Initiative. Primary Health Care System Performance in Low and Middle-Income Countries : A Rapid Scoping Review of the Evidence from 2010-2017. 2017;(June).

8. Desplats D, Kone Y, Razakarison C. [For front-line community-based general practitioners]. Med Trop (Mars). 2004;64(6):539–44.

9. Caplain R, Yacoubou I, Adedemy D, Sani A, Takam S, Desplats D. Promouvoir des soins de proximité en Afrique : l'exemple de la médecine générale communautaire au Bénin. Sante Publique. 2014;26(1 Suppl):S59-65.

10. Van Dormael M, Dugas S. La construction de la médecine de famille dans les pays en développement. W. Van Lerberghe, G. Kegels VDB, editor. Antwerp: ITGPress,; 2003.

11. Mays N, Roberts E, Popay J. Synthesising research evidence. 2001;188–220.

12. Arksey H, Malley LO. Scoping Studies: Towards a Methodological Framework. 2005;19–32.

13. Kastner M, Tricco AC, Soobiah C, Lillie E, Perrier L, Horsley T, et al. What is the most appropriate knowledge synthesis method to conduct a review? Protocol for a scoping review. BMC Med Res Methodol. 2012;12(1):1.

14. Tricco AC, Soobiah C, Antony J, Cogo E, Macdonald H, Lillie E, et al. A scoping review identifies multiple emerging knowledge synthesis methods, but few studies operationalize the method. J Clin Epidemiol. 2016;73:19–28.

15. Peters MD, Godfrey C, McInerney P, Baldini Soares C, Khalil H, Parker D. Chapter 11: Scoping reviews - JBI Wiki [Internet]. Joanna Briggs Institute Reviewer's Manual. 2017 [cited 2019 Jan 4]. Available from: https://wiki.joannabriggs.org/display/MANUAL/Chapter+11%3A+Scoping+reviews

16. Alliance for Health Policy and Systems Research. Evidence Synthesis for Health Policy and Systems : a Methods Guide. Langlois E V, Daniels K, Akl EA, editors. Geneva: World Health Organization; 2018.

17. Levac D, Colquhoun H, O'Brien KK. Scoping studies: Advancing the methodology. Implement Sci. 2010;5(1):1–9.

Annexes

Results of the search

Database	Search strategy	Date for search	Number of results
**Medline**	Search (((("Physicians"[Mesh] OR "medical doctor" OR doctor OR “family physician*” OR “family doctor*”OR “primary care physician*” OR “primary care doctor*” OR “general doctor*” OR “general practitioner*” OR GP OR “medical officer”))) AND (("Primary Health Care"[Mesh] OR “primary health care” OR "Community Health Services"[Mesh] OR "Community Health Centers"[Mesh] OR "primary care" OR "first line" OR "first level" OR "health center*" OR “general practice” OR “family medicine” OR “family practice” OR “private” OR "Private Practice"[Mesh] OR "Private Sector"[Mesh] OR "Private Facilities"[Mesh] OR “front line” OR “front-line”))) AND (("Sub Saharan Africa" OR "Africa South of the Sahara"[Mesh] OR Angol* OR Benin* OR Botswana OR “Burkina Faso” OR Burkinabe* OR Burundi OR Cameroon* OR Cameroun* OR “Cape Verde” OR “Cape Verdean” OR “Cabo Verde” OR “Central African Republic” OR Chad* OR Tchad* OR Comoros OR Comorian OR “Democratic Republic of Congo” OR “Republic of Congo” OR Congo OR Congolese OR RDC OR DRC OR Zaire OR “Republique Democratique Congo” OR “Côte d’Ivoire” OR “Republic of Côte d’Ivoire” OR “Ivory Coast” OR Ivorian OR Djibouti* OR “Equatorial Guinea” OR Guinea* OR Eritrea* OR Ethiopia* OR Gabon* OR Gambia* OR Ghana* OR Guinea OR “Guinea-Bissau” OR Kenya* OR Lesotho OR Liberia* OR Libya* OR Madagascar OR Malawi* OR Mali OR Malian OR Malien OR Mauritania* OR Mauritius OR Mauritian OR Mozambique OR Mozambican OR Namibia* OR Niger* OR Nigeria* OR Rwanda* OR “Sao Tome Principe” OR “Sao Tomean” OR Senegal* OR Seychell* OR “Sierra Leon*” OR Somali* OR “South Africa” OR “South African” OR Sudan* OR Swaziland Or Swazi OR Eswatini OR Tanzania* OR Togo* OR Uganda* OR Zambia* OR Zimbabwe*)) Filters: Publication date from 2000/01/01 to 2019/04/30; English; French	**06 April 2019**	**1510**
**Banque de données en santé publique (BDSP) de l’Ecole des hautes études en santé publique (EHESP)**<br><br>**Periode limited from 2000 to 2019**	(medecin OU "medecin generaliste" OU "medecin famille") AND ("premiere ligne" OU "Soins santé primaire " OU "prive" OU "Médecine générale" OU "sante communautaire") AND ("Afrique subsaharienne" OU "Afrique du Sud" OU Botswana OU Lesotho OU Namibie OU "République sud africaine" OU Swaziland OU Cameroun OU Centrafrique OU Congo OU "Congo Brazzaville" OU Gabon OU "Guinée équatoriale" OU "République démocratique Congo" OU Zaïre OU RDC OU Burundi OU Kenya OU Ouganda OU Rwanda OU Tanzanie OU "Cap Vert" OU Gambie OU Guinée Ou "Guinée Bissau" OU "Guinée Conakry" OU Guinée OU Libéria Ou Sénégal OU "Sierra Leone" OU "Burkina Faso" OU Mali OU Niger Ou Tchad OU Angola OU Malawi OU Mozambique OU Zambie OU Zimbabwe OU Bénin OU Côte d'Ivoire Ou Ghana OU Nigeria OU Togo OU Comores OU "Ile Maurice" OU Madagascar OU Maurice OU Seychelles OU Djibouti OU Erythrée OU Ethiopie OU Lybie OU Mauritanie OU "Sao tome et principes" OU Somalie OU Soudan) .	**06 April 2019**	**44**

**Cochrane Libray (from january 200 to may 2019**	(Physicians OR "medical doctor" OR doctor OR “family physician*” OR “family doctor*” OR “primary care physician*” OR “primary care doctor*” OR “general doctor*” OR “general practitioner*” OR GP OR "medical officer") AND ("Primary Health Care" OR "Community Health Services" OR "Community Health Centers" OR "primary care" OR "first line" OR "first level" OR "health center*" OR “general practice” OR “family medicine” OR “family practice” OR “private” OR "Private Practice" OR "Private Sector" OR "Private Facilities") AND ("Sub Saharan Africa" OR "Africa South of the Sahara" OR Angol* OR Benin* OR Botswan* OR “Burkina Faso” OR Burkinabe OR Burund* OR Cameroon* OR Cameroun* OR “Cape Verde” OR “Cape Verdean” OR “Cabo Verde” OR “Central African Republic” OR Chad* OR Tchad* OR Comoros OR Comorian OR “Democratic Republic of Congo” OR “Republic of Congo” OR Congo OR Congolese OR RDC OR DRC OR Zaire OU “Republique Democratique Congo” OR “Côte d’Ivoire” OR “Republic of Côte d’Ivoire” OR “Ivory Coast” OR Ivorian OR Djibouti* OR “Equatorial Guinea” OR Guinean OR Eritrea* OR Ethiopia* OR Gabon* OR Gambia* OR Ghana* OR Guinea OR “Guinea-Bissau” OR Kenya OR Kenyan OR Lesotho OR Liberia* OR Libya* OR Madagasca* OR Malawi* OR Mali OR Malian OR Malien OR Mauritania* OR Mauritius OR Mauritian OR Mozambique OR Mozambican OR Namibia* OR Niger* OR Nigeria* OR Rwanda* OR “Sao Tome Principe” OR “Sao Tomean” OR Senegal* OR Seychell* OR “Sierra Leon*” OR Somali* OR “South Africa” OR “South African” OR Sudan* OR Swaziland Or Swazi OR Eswatini OR Tanzania* OR Togo* OR Uganda* OR Zambia* OR Zimbabwe*)	**06 April 2019**	**447**
**Health Systems Evidence**	(Physicians OR "medical doctor" OR “medical officer” OR doctor OR “family physician*” OR “family doctor*” OR “primary care physician*” OR “primary care doctor*” OR “general doctor*” OR “general practitioner*” OR GP) AND ("Primary Health Care" OR "Community Health Services" OR "Community Health Centers" OR "primary care" OR "first line" OR "first level" OR “front line” OR "health center*" OR “general practice” OR “family medicine” OR “family practice” OR “private” OR "Private Practice" OR "Private Sector" OR "Private Facilities") AND ("Sub Saharan Africa" OR "Africa South of the Sahara")	**06 April 2019**	**151**
**Web of Science core collection**	(ALL= ("Physicians" OR "medical doctor" OR doctor OR “family physician*” OR “family doctor*” OR “primary care physician*” OR “primary care doctor*” OR “general doctor*” OR “general practitioner*” OR GP OR “medical officer”)) AND LANGUAGE: (English OR French)<br>**AND**<br>(All= ("Primary Health Care" OR "Community Health Services" OR "Community Health Centers" OR "primary care" OR "first line" OR "first level" OR "health center*" OR “general practice” OR “family medicine” OR “family practice” OR “private” OR "Private Practice" OR "Private Sector" OR "Private Facilities" OR “front line” OR “front-line”)) AND LANGUAGE: (English OR French)<br>**AND**<br>(ALL= (Namibia* OR Niger* OR Nigeria* OR Rwanda* OR “Sao Tome Principe” OR “Sao Tomean” OR	**12 April 2019**	**1685**

	Senegal* OR Seychell* OR “Sierra Leon*” OR Somali* OR “South Africa” OR “South African” OR Sudan* OR Swaziland Or Swazi OR Eswatini OR Tanzania* OR Togo* OR Uganda* OR Zambia* OR Zimbabwe*)) AND LANGUAGE: (English OR French) OR (ALL= (Chad* OR Tchad* OR Comoros OR Comorian OR Democratic Republic Congo OR Republic Congo OR Congo OR Congolese OR RDC OR DRC OR Zaire OR Republique Democratique Congo OR Côte dIvoire OR Republic Côte dIvoire OR Ivory Coast OR Ivorian OR Djibouti* OR Equatorial Guinea OR Guinea* OR Eritrea* OR Ethiopia* OR Gabon* OR Gambia* OR Ghana* OR Guinea OR Guinea-Bissau OR Kenya* OR Lesotho OR Liberia* OR Libya* OR Madagascar OR Malawi* OR Mali OR Malian OR Malien OR Mauritania* OR Mauritius OR Mauritian OR Mozambique OR Mozambican)) AND LANGUAGE: (English OR French) OR (ALL= ("Sub Saharan Africa" OR "Africa South of the Sahara" OR Angol* OR Benin* OR Botswana OR “Burkina Faso” OR Burkinabe* OR Burundi OR Cameroon* OR Cameroun* OR “Cape Verde” OR “Cape Verdean” OR “Cabo Verde” OR “Central African Republic”)) AND LANGUAGE: (English OR French))<br>**Refined by**: [excluding] **WEB OF SCIENCE CATEGORIES**: (PLANT SCIENCES OR THERMODYNAMICS OR ENERGY FUELS OR ENGINEERING MECHANICAL OR FORESTRY OR GREEN SUSTAINABLE SCIENCE TECHNOLOGY OR BIOCHEMISTRY MOLECULAR BIOLOGY OR SOIL SCIENCE OR VETERINARY SCIENCES OR AGRONOMY OR BIODIVERSITY CONSERVATION OR HISTORY OR LINGUISTICS OR AGRICULTURE DAIRY ANIMAL SCIENCE OR AREA STUDIES OR NUCLEAR SCIENCE TECHNOLOGY OR OCEANOGRAPHY OR ZOOLOGY OR FOOD SCIENCE TECHNOLOGY OR BUSINESS OR ASTRONOMY ASTROPHYSICS)<br>**Timespan**: 2000-2019. Indexes: SCI-EXPANDED, SSCI, A&HCI, ESCI.		
SciELO Citation Index	(TS= (("Physicians" OR "medical doctor" OR doctor OR family physician* OR family doctor* OR primary care physician* OR primary care doctor* OR general doctor* OR general practitioner* OR GP OR medical officer) AND ("Primary Health Care" OR "Community Health Services" OR "Community Health Centers" OR "primary care" OR "first line" OR "first level" OR "health center*" OR general practice OR family medicine OR family practice OR private OR "Private Practice" OR "Private Sector" OR "Private Facilities" OR front line OR front-line) AND (Namibia* OR Niger* OR Nigeria* OR Rwanda* OR Sao Tome Principe OR Sao Tomean OR Senegal* OR Seychell* OR Sierra Leon* OR Somali* OR South Africa OR South African OR Sudan* OR Swaziland Or Swazi OR Eswatini OR Tanzania* OR Togo* OR Uganda* OR Zambia* OR Zimbabwe* OR Chad* OR Tchad* OR Comoros OR Comorian OR Democratic Republic Congo OR Republic Congo OR Congo OR Congolese OR RDC OR DRC OR Zaire OR Republique Democratique Congo OR Côte dIvoire OR Republic Côte dIvoire OR Ivory Coast OR Ivorian OR Djibouti* OR Equatorial Guinea OR Guinea* OR Eritrea* OR Ethiopia* OR Gabon* OR Gambia* OR Ghana* OR Guinea OR Guinea-Bissau OR Kenya* OR Lesotho OR Liberia* OR Libya* OR Madagascar OR Malawi* OR Mali OR Malian OR Malien OR Mauritania* OR Mauritius OR Mauritian OR Mozambique OR Mozambican OR "Sub Saharan Africa" OR "Africa South of the Sahara" OR Angol* OR Benin* OR Botswana OR Burkina Faso OR Burkinabe* OR Burundi OR Cameroon* OR Cameroun* OR	**12 April 2019**	**102**

	Cape Verde OR Cape Verdean OR Cabo Verde OR Central African Republic))) AND LANGUAGE: (English OR French)<br><br>OR<br><br>(TI= (("Physicians" OR "medical doctor" OR doctor OR family physician* OR family doctor* OR primary care physician* OR primary care doctor* OR general doctor* OR general practitioner* OR GP OR medical officer) AND ("Primary Health Care" OR "Community Health Services" OR "Community Health Centers" OR "primary care" OR "first line" OR "first level" OR "health center*" OR general practice OR family medicine OR family practice OR private OR "Private Practice" OR "Private Sector" OR "Private Facilities" OR front line OR front-line) AND (Namibia* OR Niger* OR Nigeria* OR Rwanda* OR Sao Tome Principe OR Sao Tomean OR Senegal* OR Seychell* OR Sierra Leon* OR Somali* OR South Africa OR South African OR Sudan* OR Swaziland Or Swazi OR Eswatini OR Tanzania* OR Togo* OR Uganda* OR Zambia* OR Zimbabwe* OR Chad* OR Tchad* OR Comoros OR Comorian OR Democratic Republic Congo OR Republic Congo OR Congo OR Congolese OR RDC OR DRC OR Zaire OR Republique Democratique Congo OR Côte dIvoire OR Republic Côte dIvoire OR Ivory Coast OR Ivorian OR Djibouti* OR Equatorial Guinea OR Guinea* OR Eritrea* OR Ethiopia* OR Gabon* OR Gambia* OR Ghana* OR Guinea OR Guinea-Bissau OR Kenya* OR Lesotho OR Liberia* OR Libya* OR Madagascar OR Malawi* OR Mali OR Malian OR Malien OR Mauritania* OR Mauritius OR Mauritian OR Mozambique OR Mozambican OR "Sub Saharan Africa" OR "Africa South of the Sahara" OR Angol* OR Benin* OR Botswana OR Burkina Faso OR Burkinabe* OR Burundi OR Cameroon* OR Cameroun* OR Cape Verde OR Cape Verdean OR Cabo Verde OR Central African Republic))) AND LANGUAGE: (English OR French)<br><br>**Refined by:** [excluding] **SciELO Categories**: (AGRICULTURE DAIRY ANIMAL SCIENCE OR BUSINESS OR BUSINESS FINANCE OR RELIGION OR VETERINARY SCIENCES)<br>Indexes=SCIELO **Timespan**=2000-2019		
Literature from experts and other stakeholders in the field of family medicine and / or research in primary health care		**Various dates**	**60**
