# Supplementary material for: The expanding movement of primary care physicians operating at the first line of healthcare delivery systems in sub-Saharan Africa: A scoping review
Source: PLoS One. 2021 Oct 22;16(10):e0258955. doi: 10.1371/journal.pone.0258955 (PMC8535187; doi:10.1371/journal.pone.0258955)
Supplement: S1 Table — (DOCX) [file pone.0258955.s003.docx]

Search strategy on Medline

| Search terms | (((("Physicians"[Mesh] OR "medical doctor" OR doctor OR “family physician*” OR “family doctor*”OR “primary care physician*” OR “primary care doctor*” OR “general doctor*” OR “general practitioner*” OR GP OR “medical officer”))) AND (("Primary Health Care"[Mesh] OR “primary health care” OR "Community Health Services"[Mesh] OR "Community Health Centers"[Mesh] OR "primary care" OR "first line" OR "first level" OR "health center*" OR “general practice” OR “family medicine” OR “family practice” OR “private” OR "Private Practice"[Mesh] OR "Private Sector"[Mesh] OR "Private Facilities"[Mesh] OR “front line” OR “front-line”))) AND (("Sub Saharan Africa" OR "Africa South of the Sahara"[Mesh] OR Angol* OR Benin* OR Botswana OR “Burkina Faso” OR Burkinabe* OR Burundi OR Cameroon* OR Cameroun* OR “Cape Verde” OR “Cape Verdean” OR “Cabo Verde” OR “Central African Republic” OR Chad* OR Tchad* OR Comoros OR Comorian OR “Democratic Republic of Congo” OR “Republic of Congo” OR Congo OR Congolese OR RDC OR DRC OR Zaire OR “Republique Democratique Congo” OR “Côte d’Ivoire” OR “Republic of Côte d’Ivoire” OR “Ivory Coast” OR Ivorian OR Djibouti* OR “Equatorial Guinea” OR Guinea* OR Eritrea* OR Ethiopia* OR Gabon* OR Gambia* OR Ghana* OR Guinea OR “Guinea-Bissau” OR Kenya* OR Lesotho OR Liberia* OR Madagascar OR Malawi* OR Mali OR Malian OR Malien OR Mauritania* OR Mauritius OR Mauritian OR Mozambique OR Mozambican OR Namibia* OR Niger* OR Nigeria* OR Rwanda* OR “Sao Tome Principe” OR “Sao Tomean” OR Senegal* OR Seychell* OR “Sierra Leon*” OR Somali* OR “South Africa” OR “South African” OR Sudan* OR Swaziland Or Swazi OR Eswatini OR Tanzania* OR Togo* OR Uganda* OR Zambia* OR Zimbabwe*)) |
| --- | --- |
| Publication dates | 01 January 2000 to 30 April 2019 |
| Languages | English, French |
