# Supplementary material for: The expanding movement of primary care physicians operating at the first line of healthcare delivery systems in sub-Saharan Africa: A scoping review
Source: PLoS One. 2021 Oct 22;16(10):e0258955. doi: 10.1371/journal.pone.0258955 (PMC8535187; doi:10.1371/journal.pone.0258955)
Supplement: S3 Table — (DOCX) [file pone.0258955.s005.docx]

| **Title of the study** | **First author** | **Year** | **Country** | **Language** | **Names assigned to PCPs in the paper** | **Type of resource** | **Methodology** |
| --- | --- | --- | --- | --- | --- | --- | --- |
| Promotion of community-based care in Africa: example of community general practice in Benin | Caplain R. | 2014 | Benin | French | Médecin généraliste communautaire | Report of an experience | Case study |
| Prise en charge des hypertendus dans la ville de Cotonou (Bénin) en 2011: connaissances attitudes et pratiques des médecins généralistes | Houenassi M D | 2016 | Benin | French | Médecin généraliste | Research Article | cross-sectional study |
| Development of Family Medicine training in Botswana:Views of key stakeholders in Ngamiland | Ogundipe R M | 2015 | Bostwana | English | Family physician | Research Article | cross-sectional study |
| Choosing between nurse-led and medical doctor-led from private for-profit versus non-for-profit health facilities: A household survey in urban Burkina Faso. | Beogo I. | 2018 | Burkina Faso | English | Medical doctor | Research Article | cross-sectional study |
| Les médecins prestataires à la première ligne des soins dans la ville de Kisangani en République Démocratique du Congo : vers une typologie | Bosongo S. | 2019 | DR Congo | French | Médecin | Research Article | cross-sectional study |
| Family Medicine needs assessment: Studying the clinical work of general practitioners in Ethiopia | Philpott J | 2014 | Ethiopia | English | General practitioner | Research Article | Mixed Methods |
| Key informants’ perspectives on development of family medicine training programs in Ethiopia | Gossa W. | 2016 | Ethiopia | English | Family physician | Research Article | Qualitative study |
| Emergence of family medicine in Ethiopia [corrected]: an international collaborative education model. | Franey C. | 2016 | Ethiopia | English | Family physician | Report of an experience | Case study |
| Perceived competency deficits and challenges of family medicine trainees in sub-Saharan Africa. | Yakubu K. | 2016 | Ethiopia, Kenya, Tanzania, Uganda, Ghana, Nigeria | English | Family physician | Research Article | cross-sectional study |
| Country profile on family medicine and primary health care in Ghana | Lawson H. J.O. | 2016 | Ghana | English | Family physician | Research Article | case study |
| Partnerships Creating Postgraduate Family Medicine in Kenya | Pust R | 2006 | Kenya | English | Family physician | Research Article | case study |
| What challenges hamper Kenyan family physicians in pursuing their family medicine mandate? A qualitative study among family physicians and their colleagues. | van der Voort C.T.M. | 2012 | Kenya | English | Family physician | Research Article | Qualitative study |
| Evolution of Family Medicine in Kenya (1990s to date): a case study | Chege PM | 2016 | Kenya | English | Family doctors | Research Article | Case study |
| Current status of family medicine in Kenya; family physicians’ perception of their role | Momanyi K | 2020 | Kenya | English | Family physician | Research Article | Mixed Methods |
| Adhésion des médecins à la politique nationale de lutte contre le paludisme cinq ans après sa révision : situation à Antananarivo (Madagascar) | Andrianasolo R.L | 2012 | Madagascar | French | Médecin | Research Article | cross-sectional study |
| Prescription des médecins dans une diarrhée aiguë du nourrisson à Antananarivo, Madagascar | Ravelomanana L. | 2018 | Madagascar | French | Médecin généraliste | Research Article | cross-sectional study |
| De Tananarive au Caire : un aperçu du métier de médecin généraliste libéral dans le contexte des systèmes de santé locaux. | Jan T. | 2011 | Madagascar, tanzania, kenya, ethiopia | French | Médecin généraliste | Thesis | Multiple-case studies |
| Family medicine training and practice in Malawi: History, progress, and the anticipated role of the family physician in the Malawian health system. | Makwero M | 2017 | Malawi | English | Family physician | Litterature review | Narrative review |
| Une médecine rurale de proximité l'expérience des médecins de campagne au Mali | Coulibaly S | 2007 | Mali | French | Médecin de campagne | Report of an experience | Case study |
| Introduction des sciences des sciences sociales sociales dans une expérience de formation professionnelle des médecins de campagne campagne au Mali | Van Dormael M. | 2007 | Mali | French | Médecin de campagne | Research Article | case study |
| North–South exchange and professional development: experience from Mali and France | Van Dormael M. | 2007 | Mali | English | Rural doctor | Research Article | Qualitative study |
| Appropriate training and retention of community doctors in rural areas: a case study from Mali | Van Dormael M. | 2008 | Mali | English | Rural doctor | Research Article | Action-research |
| Évaluation du programme d'appui à la médicalisation des aires de santé rurales au Mali. Accroître l'accès aux personnels de santé dans les zones rurales ou reculées. Étude de cas N°2. | Codjia L. | 2010 | Mali | French | Médecin de campagne | Report of a research | Mixed Methods |
| La délégation des tâches à l'ère de la e-santé pour soutenir les interventions communautaires en santé maternelle et infantile : leçons apprises du projet PACT-Denbaya | Bagayoko C.O. | 2017 | Mali | French | Médecin généraliste | Research Article | Action-research |
| Etendue des problèmes de santé pris en charge par les médecins en soins primaires au Mali et en France : en attente d’une transition des pratiques en Afrique subsaharienne ? | Naville R | 2020 | Mali | French | Médecin de campagne | Thesis | cross-sectional study |
| Pour une médecine générale communautaire en première ligne. | Desplats D. | 2004 | Mali, Madagascar | French | Médecin généraliste communautaire | Report of an experience | Case study |
| Quelle formation diplômante pour promouvoir et valoriser la pratique médicale de proximité en milieu rural? L'expérience de Santé Sud | Desplats D. |  | Mali, madagascar, Benin, guinée | French | Médecin généraliste communautaire | Report of an experience | Multiple-case studies |
| La construction de la médecine de famille dans les pays en développement | Dugas S | 2003 | Mali, south Africa | French | Médecin de famille, médecin généraliste, médecin de campagne | Report of a research | Multiple-case studies |
| Stakeholders' perceptions of the delivery and quality of sexually transmitted infection treatment by private practitioners in Windhoek, Namibia | Iipinge S. N. | 2010 | Namibia | English | General practitioner | Research Article | Qualitative study |
| The Delivery and Quality of Sexually Transmitted Infections Treatment by Private General Practitioners in Windhoek Namibia | Iipinge S. N. | 2012 | Namibia | English | General practitioner | Research Article | cross-sectional study |
| The state of readiness of Lagos State Primary Health Care Physicians to embrace the care of depression in Nigeria. | Ola B. | 2013 | Nigeria | English | Primary care physician | Research Article | cross-sectional study |
| Awareness of hypertension guidelines and the diagnosis and evaluation of hypertension by primary care physicians in Nigeria. | Ale O.K. | 2017 | Nigeria | English | Primary care physician | Research Article | cross-sectional study |
| Quality of primary care physicians’ communication of diabetes self-management during medical encounters with persons with diabetes mellitus in a resource-poor country | Ojo O.S | 2018 | Nigeria | English | Primary care physician | Research Article | cross-sectional study |
| Can task-shifting work at scale?: Comparing clinical knowledge of non-physician clinicians to physicians in Nigeria | Villar Uribe M. | 2018 | Nigeria | English | Medical officer | Research Article | cross-sectional study |
| Diabetes care knowledge and practice among primary care physicians in Southeast Nigeria: a cross-sectional study | Ugwu | 2020 | Nigeria | English | Primary care physician | Research Article | cross-sectional study |
| Benzodiazepines prescription in Dakar: a study about prescribing habits and knowledge in general practitioners, neurologists and psychiatrists. | Dièye A M | 2006 | Senegal | English | Generalist | Research Article | cross-sectional study |
| Prescription des benzodiazépines par les médecins généralistes du privé à Dakar : Enquête sur les connaissances et les attitudes | Dièye A M | 2007 | Senegal | French | Médecin généraliste | Research Article | cross-sectional study |
| Notification of occupational diseases by general practitioners in the Western Cape. | Govender M. | 2000 | South Africa | English | General practitioner | Research Article | cross-sectional study |
| The performance of different models of primary care provision in Southern Africa | Mills A | 2004 | South Africa | English | General practitioner | Research Article | Mixed Methods |
| Family medicine in South Africa: where are we now and where do we want to be? | Hellenberg D. A. | 2005 | South Africa | English | Family physician | Litterature review | Narrative review |
| Developing family medicine in South africa: a new and important step for medical education. | HELLENBERG D. | 2007 | South Africa | English | Family physician | Litterature review | Narrative review |
| Addressing alcohol problems in primary care settings: a study of general medical practitioners in Cape Town, South Africa. | Koopman F. A. | 2008 | South Africa | English | General practitioner | Research Article | cross-sectional study |
| How much is not enough ? Human resources requirements for primary health care : a case study from South Africa. TT - Combien en manque-t-il ? Besoins en ressources humaines pour les soins de santé primaire : étude de cas menée en Afrique du Sud. | Daviaud E. | 2008 | South Africa | English | Doctor | Research Article | Modelisation and stakeholders consultations |
| Access to and use of the Internet by South African general practitioners | Masters K. | 2008 | South Africa | English | General practitioner | Research Article | cross-sectional study |
| Reflections on the development of family medicine in the Western Cape: a 15-year review | Mash B. | 2011 | South Africa | English | Family physician | Research Article | case study |
| General practitioners' perceptions on management of epilepsy in primary care settings in Cape Town, South Africa: an exploratory pilot study. | Keikelame M. J. | 2012 | South Africa | English | General practitioner | Research Article | qualitative study |
| Assessment of the impact of family physicians in the district health system of the Western Cape, South Africa. | Swanepoel M. | 2014 | South Africa | English | Family physician | Research Article | Qualitative study |
| Implementation of the principles of primary health care in a rural area of South Africa | Visagie S. | 2014 | South Africa | English | Doctor | Research Article | qualitative study |
| The views of key leaders in South Africa on implementation of family medicine: critical role in the district health system. | Moosa S. | 2014 | South Africa | English | Family physician | Research Article | Qualitative study |
| Development of a family physician impact assessment tool in the district health system of the Western Cape Province, South Africa. | Paiso KS | 2014 | South Africa | English | Family physician | Research Article | Mixed Methods |
| A situational analysis of training for behaviour change counselling for primary care providers, South Africa | Malan Z. | 2015 | South Africa | English | Family physician | Research Article | Qualitative study |
| How far does family physician supply correlate with district health system performance? | Dyers R. E. | 2015 | South Africa | English | Family physician | Research Article | ecological study |
| Strengthening primary health care through primary care doctors: the design of a new national Postgraduate Diploma in Family Medicine | Mash R. | 2015 | South Africa | English | Primary care doctors | Report of an experience | Case study |
| The roles and training of primary care doctors: China, India, Brazil and South Africa. | Mash R. | 2015 | South Africa | English | Primary care doctors | Research Article | Qualitative study |
| The self-reported learning needs of primary care doctors in South Africa: a descriptive survey | Malan Z. | 2015 | South Africa | English | Medical officer, general practitionner | Research Article | cross-sectional study |
| Leadership and governance: learning outcomes and competencies required of the family physician in the district health system | Mash R. | 2016 | South Africa | English | Family physician | Report of an experience | Case study |
| Examining the influence of family physician supply on district health system performance in South Africa: An ecological analysis of key health indicators | Von Pressentin K. B. | 2017 | South Africa | English | Family physician | Research Article | Retrospective longitudinal study |
| Laboratory test result interpretation for primary care doctors in South Africa | Vanker N. | 2017 | South Africa | English | Primary care doctors | Research Article | cross-sectional study |
| Reaching national consensus on the core clinical skill outcomes for family medicine postgraduate training programmes in South Africa. | Akoojee Y. | 2017 | South Africa | English | Family physician | Research Article | Delphi study |
| The bird’s-eye perspective: how do district health managers experience the impact of family physicians within the South African district health system? A qualitative study | Von Pressentin KB | 2017 | South Africa | English | Family physician | Research Article | Qualitative study |
| Baseline measures of primary health care team functioning and overall primary health care performance at Du Noon Community Health Centre. | Mukiapini S. | 2018 | South Africa | English | Family physician; Doctor | Research Article | Mixed Methods |
| The perceived impact of family physicians on the district health system in South Africa: a cross-sectional survey. | Von Pressentin K. B. | 2018 | South Africa | English | Family physician | Research Article | cross-sectional study |
| Training of workplace-based clinical trainers in family medicine, South Africa: Before-and-after evaluation. | Mash R. | 2018 | South Africa | English | Family physician | Research Article | Mixed Methods |
| The Influence of Family Physicians Within the South African District Health System: A Cross-Sectional Study | Von Pressentin KB | 2018 | South Africa | English | Family physician | Research Article | cross-sectional study |
| How well do public sector primary care providers function as medical generalists in Cape Town: a descriptive survey | Christoffels R. | 2018 | South Africa | English | Medical officer | Research Article | cross-sectional study |
| The impact of family physician supply on district health system performance, clinical processes and clinical outcomes in the Western Cape Province, South Africa (2011–2014) | Chinhoyi R. L. | 2018 | South Africa | English | Family physician, medical officer | Research Article | Retrospective longitudinal study |
| Strengthening the district health system through family physicians | Mash R. | 2018 | South Africa | English | Family physician | Litterature review | Narrative review |
| Exploring the sorting of patients in community health centres across Gauteng Province, South Africa. | Stott B. A. | 2019 | South Africa | English | Primary health care doctor | Research Article | qualitative study |
| Primary healthcare delivery models for uninsured lowincome earners during the transition to National Health Insurance: Perspectives of private South African providers | Girdwood, S | 2019 | South Africa | English | General practitioner | Research Article | Multiple-case studies |
| Exploring the beliefs and attitudes of private general practitioners towards national health insurance in Cape Town, South Africa | Mathew S | 2019 | south Africa | English | General practitioner | Research Article | Qualitative study |
| Where are they working? A case study of twenty Cuban-trained South African doctors | Motala M | 2019 | South Africa | English | Médical officer, gps | Research Article | Qualitative study |
| A human resources for health analysis of registered family medicine specialists in South Africa: 2002–19 | Tiwari R | 2020 | South Africa | English | Family Physician, medical practitioner | Research Article | Retrospective longitudinal study |
| Exploring the key principles of Family Medicine in sub Saharan Africa international Delphi consensus process | Mash R. | 2008 | South Africa, Democratic Republic of the Congo, Uganda, Kenya and Tanzania | English | Family physician | Research Article | Delphi study |
| Perspectives on key principles of generalist medical practice in public service in sub-saharan africa: a qualitative study | Reid S | 2011 | South Africa, Lesotho, Botswana, Swaziland, Kenya, Uganda, Rwanda and Ghana. | English | Medical officer | Research Article | Qualitative study |
| Understanding of family medicine in Africa: a qualitative study of leaders’ views | Moosa S. | 2013 | SSA | English | Family physician | Research Article | Qualitative study |
| Developing family practice to respond to global health challenges: The Besrour Papers: a series on the state of family medicine in the world. | Arya N | 2017 | SSA | English | Family physician | Litterature review | Narrative review |
| Family medicine around the world: overview by region: The Besrour Papers: a series on the state of family medicine in the world. | Arya N | 2017 | SSA | English | Family physician | Litterature review | Narrative review |
| A scoping review on family medicine in sub-Saharan Africa: practice, positioning and impact in African health care systems | Flinkenflögel M | 2020 | SSA | English | Family physician | Litterature review | Scoping review |
| Confidence in Procedural Skills before and after a Two-YearMaster’s Programme in Family Medicine in Gezira State, Sudan | Mohamed K. G. | 2017 | Sudan | English | Family physician | Research Article | before and after study |
| Family medicine's role in health care systems in Sub-Saharan Africa: Uganda as an example. | Ssenyonga R | 2007 | Uganda | English | Medical officer | Litterature review | Narrative review |
| Roles and challenges of family physicians in Uganda: A qualitative study | Besigye I. K. | 2019 | Uganda | English | Family physician | Research Article | Qualitative study |
