# Supplementary material for: The expanding movement of primary care physicians operating at the first line of healthcare delivery systems in sub-Saharan Africa: A scoping review
Source: PLoS One. 2021 Oct 22;16(10):e0258955. doi: 10.1371/journal.pone.0258955 (PMC8535187; doi:10.1371/journal.pone.0258955)
Supplement: Attachment — Submitted filename: PLOS ONE-D-20-25494 Review_2020_11_03.pdf [file pone.0258955.s006.pdf]

PONE-D-20-25494 Review

General: This manuscript is the write up of a scoping review that was conducted to examine "…the current literature on First Line Medical Doctors (FLMD) in Sub-Saharan Africa (SSA)…" in order to "identify the knowledge gaps" (p.2 Protocol) about "what are the main characteristics and key issues of FLMD practices in SSA" (lines 92-93). Reviewing the literature over the past 19 years (2000-2019) in both English and French using a number of pre-identified search terms in five databases and purposively sampling some grey literature, the authors include 73 peer-reviewed (or original) research publications in their final analysis. The results indicate a range of publications, largely from South Africa (47%), and written in English (67%), although there are a number of papers (29%) from French-speaking Africa. There is great heterogeneity in the nomenclature of these "first line" doctors across SSA; and, the authors propose a bilingual classification based on how the literature refers to them. The authors subsequently construct three categories of FLMDs after examining their scope of practice and responsibilities whether in Anglophone Africa or in *la Francophonie*. Going systematically across four components of the health system (professional identity; governance; roles and activities; and, output and outcomes) for each type of FLMD (Family Physician; *medicin generalist communautaire*; or General Practitioner) the study reveals interesting observations about how, where, why and under what conditions these doctors work. These findings have important repercussions for national and regional health systems planners in Africa as well as newly qualified doctors deciding on career pathways. The paper also exposes significant gaps in research on medical doctors who function at primary care level, especially those in private practices across SSA and in rural areas as solo practitioners (as opposed to those placed there as part of government and/or NGO programmes). There is also little understanding of the return on investment in this cadre of health care worker *vs* the risks.

In summary, this article provides a refreshing look at task shifting in reverse and posits that when medical doctors move from referral centres into primary care spaces across Sub-Saharan Africa, there may be unforeseen consequences that could be managed with better role definition, governance and policies. It is critically important to understand the phenomenon of a shift of doctors away from referral hospitals into communities and clinical spaces while attending to undifferentiated patients at the primary care level, especially as these doctors engage patients and communities at points of first contact that were historically reserved for nurses. There are also potential policy decisions in resource constrained environments, since training and upskilling medical doctors to occupy roles in primary health care represents a costly investment. This is arguably a novel and relevant topic for a scoping review, and potentially can make substantive contributions towards addressing some of the human resources for health challenges, as well as the political and ethical ones.

Finally, this paper represents an ambitious project; and, the amount of work and reflection are obvious. The original research protocol is included as supplemental material, and indicates that this is part of a PhD from a candidate in Benin being supervised in Belgium. It is important for ISI and mainstream medical journals to publish articles about Africa, especially those conceived and written by Africans.

Major: There are several significant conceptual framings to the manuscript and methodological decisions that, to my mind, require clearer explanation and more rigorous justification in the text. I

have outlined these in the first three points below, followed by other major issues in no particular rank order of importance:

1. Including a more robust definition of the “first-line medical doctor” (FLMD) in the Introduction is critical. Although FLMDs are the cornerstone of this study, this is not a common term in the medical literature, nor is it one with which most readers (even English-speaking medical generalists) would be familiar. A clear definition of FLMDs will enable the article to reach the broader medical community and articulate with conversations in other journals about the deployment of primary care doctors in SSA and their scope of practice. To illustrate the limitation of the use of FLMD: when I tried to search within the PLOS ONE option for similar publications on MEDLINE, this is the message I received: “Your search was processed without automatic term mapping because it retrieved zero results.” Similarly, when I used "first line medical doctor" in PUBMED on 2020/10/25, this is the response I got: “Quoted phrase not found.” In Google scholar (albeit not ideal), there were eight results (five unique, three duplicates, one from Quebec, the rest European). For a predominantly English audience, this term needs more unpacking, and will probably necessitate revision of the title of the manuscript to in order to resonate with a wider (and more appropriate) audience.

2. There is an uneasy linkage between FLMDs in the manuscript (whose role is largely clinical) and the pillars of health systems research. I could not find sufficient justification for the use of the health systems dynamics framework (Reference 27) as the sole basis for categorising the articles and creating a data extraction form (lines 117 – 120). Since the data extraction form does not appear to be included in either the body of the paper or in the supplemental files, it is unclear exactly what data were extracted from which articles onto an Excel spreadsheet. This omission also speaks to the limited availability of data, which is something PLOS ONE requires. Furthermore, it seems that this framework gets jettisoned later in the Discussion and Conclusion, failing to take full advantage of the model in its ability “to deal with complexity/its dynamic character/and the values embedded in it.” There is no convincing explanation as to why the framework was employed to begin with, since “health systems” is not included in the key words, nor were these different components (“governance”; “resources”; “service delivery”; “relationships with the community context”; “values on which the health system is based”; and, “outcomes”) used in the search strategy at the outset. Rather than allowing the themes of the scoping review to emerge organically and synergistically through iterative discussions among the authors (as it seems might have been the case anyway in Table 5), the method of using the ‘health systems dynamics’ framework could have restricted the reviewers’ vision of appreciating the actual subject matter being covered by these 73 articles. Without access to the Excel data extraction form, and only the S3 Table (List of papers included), it is difficult to understand the data items and the data charting process. As a reviewer, it would have been important to confirm the fields on the data extraction form, and look for alignment on several of the articles through a trial run. At the moment, none of this is replicable. Being familiar with some of the literature, it seems that several important (and statistically significant) outcomes were not adequately captured.

3. Aside from not using any of the key components of the 'health systems dynamics' framework above in the search strategy, such as "service delivery", there seem to be other aspects of the search strategy that are not clear or do not align with the stated objectives of the scoping review extracted below from the Protocol (S1 Text):
   - *This scoping review aims at […] providing an overview of the current literature on First Line Medical Doctors (FLMD) in Sub-Saharan Africa and identifying the knowledge gaps.* (p 2 Protocol)
   - *General objective: the general objective is to map the existing knowledge on First Line Medical Doctors (FLMD) in Sub-Saharan Africa and to identify the key issues in this area.*
   - *Specific objectives and research questions:*
     a. Determine the various types of medical Doctors operating at the level of first-line in SSA
     b. Map the key dimensions that have been studied on FLMD in Sub-Saharan Africa (training, resources, services delivery, leadership and government [sic] arrangements, etc.)
     c. Determine the key issues related to FLMDs' practice in Sub-Saharan Africa

   My specific questions in this regard are:

   3.1 While the justification for starting the search from the year 2000 is based on the prevalence of articles dating from that year (line 102), there may be foundational publications that could have been missed. Having been part of early debates that dealt with questions of professional identity and the need for a values-driven medical practice at primary care level in SSA ("one family"/ "one doctor"), it seems prudent to go back to the 1990's, or provide a stronger justification for limiting the search to publications dating from 2000 other than convenience. Even if the year 2000 is retained, this decision could be dealt with in the Discussion, with reference to earlier seminal articles not included in the scoping review, in order to apply a historical/ contextual dimension and evolutionary analysis to what has been published over this nearly 20 year period and how it has developed over time.
   3.2 Why was there a decision to leave out government policy documents on the deployment of FLMDs? It seems that inclusion of this type of grey literature would be important in order to answer the research question/objectives of the study. Through government documents or discussion papers, each country in SSA might have been represented in ways that publishing in peer-reviewed indexed journals with high APCs would never be possible. The authors might perhaps consider explaining this exclusion and adding it to the section on limitations.
   3.3 Also on the matter of grey literature, it is unclear as to why other NGOs (besides Sante Sud) or University Medical Faculties or professional groupings of primary care practitioners (besides PRIMAFAMED) or religious groups working in SSA were not purposively sought out. As notional examples, I am thinking of government-to-government agreements between Cuba and countries in SSA to supply first line doctors; MSF and other humanitarian relief agencies; CHAI support to the training of medical doctors in Rwanda; etc.
   3.4 Since the colonisation of SSA was not restricted to the English and the French, why was the search not trilingual to include Portuguese, or even other languages such as

German, Dutch and Flemish? There would be many SSA countries with such colonial histories, and legacies of these relationships, whose publications might have been missed. Again, perhaps something to include in limitations?

3.5 I am curious as to why an expanded search was not undertaken once certain framings came to light through the analysis of the articles. For example, after the French terms *medecin de campagne* or *generaliste communautaire* came up, why were the English translations/interpretations not subsequently incorporated into the search strategy? "Rural doctor" yields a great deal of results that do not appear to be included in the 73 articles. Likewise, the literature on "community service doctors" in the South African context, and perhaps others, is missing, although it appears similar to the *generaliste communautaire*.

3.6 As well, in the search strategy, it is not clear why a manual review of several journals critical to this subject matter was not undertaken. For example, purposive sampling of *Rural and Remote Health*, *African Journal of Primary Care and Family Medicine* and *Human Resources for Health* might have yielded results that were more focussed and/or relevant to the key questions of the scoping review.

3.7 Finally, the correct reference for the PRISMA extension for Scoping Reviews (reference 26) should be Tricco AC, Lillie E, Zarin W, O'Brien KK, Colquhoun H, Levac D, et al. PRISMA Extension for Scoping Reviews (PRISMAScR): Checklist and Explanation. Ann Intern Med; 169:467–473. doi: 10.7326/M18-0850. Although the correct PRISMA-ScR document is included as "S2 Text", it is still missing the appropriate citation in the body of the manuscript (JBI has taken down the link in #26) as well as permission to re-print from St Michael's. Using Tricco et al as a guide will assist in revising the above points as well as addressing some of what follows below.

4. The Flow Chart, Figure 1, is missing critical information that makes it difficult for the reader to follow why some articles were excluded at certain points and why others were retained. In addition, aside from a brief mention of the review process in the lines 131-132, a more robust narrative should be included in the Results section to describe the flow chart.

   Specifically:
   4.1 The disaggregated sources of articles should be included in the "Identification" step, with the exact numbers sourced from each of the five databases, rather than a combined total of n=3939. "Other sources" should also be itemised at this point.

   4.2 The reasons for excluding 3844 records through screening the title and abstract are not clearly articulated in either the text or in the flow diagram. Lines 112-113 indicate, that "after removing the duplicates, two of the authors independently assessed the titles, abstracts, and full-text of the articles with predefined selection criteria (Table 2)", thereby appearing to merge the steps of full-text review ("Eligibility") with that of "Screening". This distinction needs to be maintained, rather than simply stating that 3844 records were excluded without providing a clear rationale or criteria for elimination.

   4.3 The 70 articles that were excluded based on the eligibility criteria might be valid; however, the restrictions are not completely aligned with what is outlined in Table 2. For example, one of the exclusion criteria is "papers published before 2000" whereas in the flow

diagram, manuscripts were excluded because “data [were] collected before 2000”. The reasons (inclusion/exclusion criteria) need to be consistent and strictly adhered to throughout this process. As stated above, opinion papers, commentaries and conference or workshop reports might have been valuable to retain, given that this is a scoping (rather than a systematic) review. The focus of the scoping review on “peer reviewed” original research requires further justification, as indicated in Point 3 above. Another exclusion criterion from Table 2 “Papers relating to MDs working exclusively in hospitals” might have inadvertently eliminated research about important cadres of first line doctors, especially those working in rural areas in SSA, where there may only be a district or mission hospital serving a sparsely populated area through outreach initiatives rather than a formalised network of other PHC facilities (such as clinics or health posts).

4.4 In the “Inclusion” step, the addition of 17 articles through citation tracking and six articles through a new search should be indicated with arrows below the box of “Full-text articles eligible” (n=50), as well the exact date of the new search (not only the year). These should then be merged, to add 23 articles to the 50, for the total of 73.

4.5 Finally, although additional tables and diagrams are used in the manuscript to describe the types of articles included, it might be useful to add a basic characterisation of the types of sources that made the cut. This could go in below the “Papers included in the scoping review”, and might be a classification of the number of those written in French *vs* English, or about specific countries in SSA (therefore removing Figure 2), or by type of methodology (quantitative, qualitative and mixed methods)—to better (and more graphically) indicate the heterogeneity and variety of the final selections.

5. While the article requires copy editing to address grammatical, spelling and punctuation errors, there are important clarifications of terminology that might have gotten lost in translation from French to English. Most striking amongst these are:

5.1 In the Results section, the use of “denomination” to indicate “nomenclature” or “taxonomy” to describe the different types of FLMDs discussed in the literature. This should be addressed in lines 138 – 140; 142, and in Table 3.

5.2 Furthermore, some aspects of categorising these doctors into groupings based on the descriptions of their activities seem problematic (Figure 3). For me, combining medical officers (MOs) and GPs misses the point of general practitioners’ largely private practices being run as small businesses with pharmacies and dispensing options as well as niche or boutique foci, such as aesthetic medicine: they are inherently unregulated. Ironically, however, at least in the South African context, GP practices are stable caring for families and communities across generations. Medical officers operating at first line, on the other hand, are never self-employed, and they often work publically. Their contractual relationships are either with either government or another regulatory body, such as a health insurer or syndicate of health care providers. Like the *generalistes communautaires* (and community service doctors), MOs are usually transitory remaining in a post until an opportunity for specialisation opens up. The groupings, and conclusions subsequently drawn, do not seem plausible. Therefore, Figure 3 needs revision.

6. Discussion: The phrase “a multiform phenomenon” (line 403) needs re-wording. Care must also be taken in terms of sweeping generalisations “…in the RSA, FPs are trained for four years and work mainly in hospitals…” (lines 415 – 416). There are regional differences within RSA, with provinces deploying FPs to community health centres and/or sub-districts rather than to district hospitals. Similarly, lines 425 – 429 starting with “The FLMDs are poorly integrated into local health systems and national health policies […]” might be over-stated from the articles available. Simply because the authors “did not find a clear and overarching governance framework to guide FLMDs” does not mean that in certain contexts it does not exist. It simply means that the authors did not find it! This brings me to the point of the purposes and limitations of this type of study and the conclusions that can in fact be drawn from a scoping review. There is no critique in this manuscript about what types of research gets published and indexed from the Global South, and especially from SSA. In this review, South Africa/PRIMAFAMED/NGO Sante Sud dominate and therefore, simply put, get to tell the story. It would behove the authors to highlight the research gaps more clearly and raise issues around publication bias. Certain journals have annual quotas for articles from Africa—and perhaps other parts of the Global South. APCs are prohibitive and most journal publications are in English. Therefore, the current Conclusion over-reaches in places about what can be said from this scoping review, and should be re-framed in the tone highlighted in line 459, “…more empirical evidence is needed before drawing final conclusions.” The study can only comment about gaps in the literature that describe what is, or perhaps more importantly, what is not there.

Minor:

1. In the Introduction, referencing the increasing number of doctors across Africa per population is important but does not seem specific to the practice of primary care. To strengthen the point, it would be helpful to include the shift towards generalism (vs specialty training) of doctors (if this is true?) as well as overall statistics from SSA, rather than only Benin and the DRC.
2. There are many tables, figures, a map, supplementary materials, etc. that are rather overwhelming. I wonder whether some of these might be combined or omitted entirely? I would suggest moving Table 1 (the search strategy) into an appendix, and integrating the information from Figure 2 elsewhere. The numbers in Table 3 do not add up (total of 78), so it needs to be stated if there were multiple designations of doctors studied in a single paper. I would also recommend trying to combine information from Tables 3 and 4 with Figure 2 in some way (so the map could stay with the particular countries appropriately labelled). The fragmentation of information makes the paper seem more complex than it is and difficult to follow. Table 5 columns should be flipped: “Themes” on the left and “Dimensions” on the right. I am also not sure these are the correct labels for the headings (another French to English ‘lost in translation’?) because, as stated above, the data extraction form is not available.
3. NB: Libya not in SSA. It might be useful to indicate or reference your source(s) for all of the countries designated in your search strategy as being in SSA (eg: UNESCO; World Bank; etc.)
4. While discussing the division of labour in the manuscript (who did what), you might want to consider indicating this by including the authors’ initials.

5. Once the issues above are addressed, the abstract will require revision. Either way, the ‘PCC’ should be made clear in the Introduction; the Methods section would benefit from synthesis and alignment with the text of the manuscript; the Findings should reflect only what is included in the 73 articles under review; similarly, the Conclusion can only draw on what is published and/or identified as gaps in the literature. I am a bit worried about some of the quantitative declarations like, “the increasing presence of medical doctors at the first line of health care” or the assertion that GPs “constitute the bulk of FLMDs in SSA” when it is not clear that this information is derived from the scoping review. See comments above about possible over-stating the concluding points. Once again, a scoping review can only draw conclusions about what is already published, or comment on research gaps.
6. The term “professional identity” within medicine has been richly explored. Lines 171 – 176 would benefit from citing some of this literature, rather than the generic occupational reference #28. This is important to adequately set up what follows later regarding the “Professional Identity of FLMDs” in lines 196 – 249.
7. There is only brief mention of limitations on data quality. This could be addressed in more detail.
8. References: I am curious about why DOIs were not routinely included.
9. There are several categorical statements in the manuscript which are clearly false, or end up being contradicted later. One of these being line 237: “The GPs […] are medical doctors with a mere undergraduate training.” Unfortunately, this is simply not true. Most countries require some form of vocational training following an undergraduate degree, prior to working as a general practitioner or medical officer. This could be a rotating one or two year internship as well as an additional year of community service. As an example of conflicting information, lines 277 – 278 “…most of the MGCs work in CHCs, which are public entities…” contradicts line 224 which states “The MGCs work in the private sector and claim not-for-profit status.” Table 6 is also prone to such errors (eg: FPs “do not provide first-contact care”), and should be reviewed. A final careful read-through of the text would be important to address such discrepancies.
10. While not the focus of this current study, one wonders about the impacts of migration, brain drain, the climate crisis and regional conflicts on the subject under review. Even passing mention of these factors might be in order in the Discussion given how prevalent and weighty these are for the SSA context within which this review takes place.
