# Supplementary material for: The expanding movement of primary care physicians operating at the first line of healthcare delivery systems in sub-Saharan Africa: A scoping review
Source: PLoS One. 2021 Oct 22;16(10):e0258955. doi: 10.1371/journal.pone.0258955 (PMC8535187; doi:10.1371/journal.pone.0258955)
Supplement: Attachment — Submitted filename: Response to Reviewers.pdf [file pone.0258955.s008.pdf]

Kéfilath Bello
Centre de Recherche en Reproduction Humaine et en Démographie
kefilath@gmail.com, + 229 96 21 74 50
Carré 129 Patte d'Oie , Ahouanlèko, Maison Alabah, 01 BP1822 Cotonou, Benin

September 07, 2021

Dr Virginia E. M. Zweigenthal
Academic Editor
PLOS ONE

Subject: Rebuttal letter for manuscript PONE-D-20-25494R1

Dear Dr Zweigenthal,

We were pleased to receive the latest review of our manuscript. The suggestion to include the authors' positionality in the paper has enriched our work and further positioned it in the current global health debates. We are sincerely grateful for the quality contributions made to the paper throughout the review process. We hope that the manuscript will meet PLOS ONE's quality requirements in its current version.

Please find below the response to the unique point raised by the reviewers. We have also corrected some problems raised by the editorial office regarding the numbering of the tables.

We attach to this letter a marked-up copy of the revised manuscript that includes now a postscript on the authors' positionality. We also attached an unmarked version of the manuscript.

We remain available for any other request to improve this paper.

Sincerely,

Kéfilath Bello

Response to the reviewer 3

Review comment

One of the responses to the reviewer regarding the question of authorship to include authors from Africa was well presented in the response to the reviewer, and it would strengthen the paper if a statement to that effect either is included as 'positionality' in the manuscript or, perhaps more appropriately, as a postscript at the end of the paper. The current discussions regarding decoloniality and authorship of research from the global south in the field of global health is arguing for a more transparent positioning.

Our response

Thank you very much for reviewing our manuscript and for your valuable comment. The suggestion to include a statement on the authors' positionality was an important contribution to our work. It helped further to position the paper in the current global health debates.

As suggested, we added a postscript on the authors' positionality (see line 656 to line 705 in the revised manuscript).

Response to the issues raised by the editorial office

Sincere apologies to the issues related to the numbering of the tables. In fact, we deleted a table from the first submission. This led to this error in the numbering. Please find below how we have addressed the issues in the revised manuscript.

Issue 1
Please ensure that you refer to Table 4 and table 5 in your text as, if accepted, production will need this reference to link the reader to the Table.

Our response
We now referred to table 4 and table 5 on lines 230 and 495 respectively.

Issue 1
Please include a copy of Table 6 which you refer to in your text on page 27.
We meant to refer to table 5, not table 6. We corrected this in the revised manuscript, line 495.
